# More Prominent Inflammatory Response to Pachyman than to Whole-Glucan Particle and Oat-β-Glucans in Dextran Sulfate-Induced Mucositis Mice and Mouse Injection through Proinflammatory Macrophages

**DOI:** 10.3390/ijms23074026

**Published:** 2022-04-05

**Authors:** Pratsanee Hiengrach, Peerapat Visitchanakun, Malcolm A. Finkelman, Wiwat Chancharoenthana, Asada Leelahavanichkul

**Affiliations:** 1 Center of Excellence on Translational Research in Inflammation and Immunology (CETRII), Department of Microbiology, Chulalongkorn University, Bangkok 10330, Thailand; pratsaneeh@gmail.com (P.H.); peerapat.visitchanakun@gmail.com (P.V.); 2 Associates of Cape Cod, Inc., East Falmouth, MA 02536, USA; mfinkelman@acciusa.com; 3 Tropical Nephrology Research Unit, Department of Clinical Tropical Medicine, Faculty of Tropical Medicine, Mahidol University, Bangkok 10400, Thailand; 4 Tropical Immunology and Translational Research Unit, Department of Clinical Tropical Medicine, Faculty of Tropical Medicine, Mahidol University, Bangkok 10400, Thailand; 5 Nephrology Unit, Department of Medicine, Faculty of Medicine, Chulalongkorn University, Bangkok 10330, Thailand

**Keywords:** β-glucans, mice, proinflammatory macrophages, DSS-induced mucositis, microbiome, extracellular flux

## Abstract

(1→3)-β-D-glucans (BG) (the glucose polymers) are recognized as pathogen motifs, and different forms of BGs are reported to have various effects. Here, different BGs, including Pachyman (BG with very few (1→6)-linkages), whole-glucan particles (BG with many (1→6)-glycosidic bonds), and Oat-BG (BG with (1→4)-linkages), were tested. In comparison with dextran sulfate solution (DSS) alone in mice, DSS with each of these BGs did not alter the weight loss, stool consistency, colon injury (histology and cytokines), endotoxemia, serum BG, and fecal microbiome but Pachyman–DSS-treated mice demonstrated the highest serum cytokine elicitation (TNF-α and IL-6). Likewise, a tail vein injection of Pachyman together with intraperitoneal lipopolysaccharide (LPS) induced the highest levels of these cytokines at 3 h post-injection than LPS alone or LPS with other BGs. With bone marrow-derived macrophages, BG induced only TNF-α (most prominent with Pachyman), while LPS with BG additively increased several cytokines (TNF-α, IL-6, and IL-10); inflammatory genes (iNOS, IL-1β, Syk, and NF-κB); and cell energy alterations (extracellular flux analysis). In conclusion, Pachyman induced the highest LPS proinflammatory synergistic effect on macrophages, followed by WGP, possibly through Syk-associated interactions between the Dectin-1 and TLR-4 signal transduction pathways. Selection of the proper form of BGs for specific clinical conditions might be beneficial.

## 1. Introduction

Glucans are glucose polymers with diverse structural characteristics, including branching, molecular weight, and isomeric form (α or β), that are not synthesized by mammals [1]. While cellulose (β-1→4-glucan) is a well-known β-glucan (BG) from plants, other forms of BG are observed in microbes; yeasts; fungi; mold; seaweed; and specific plant tissues and products (oats, cereals, and barley) [2]. In a molecular structure, BG is the natural polysaccharide consisting of sequential D-glucose moieties linked by β-(1→3)-glycosidic bonds with structural varieties occurring depending on the source [3]. As examples, mushroom BG has short β-(1→6)-linked branches from the β-(1→3) backbone, while BG of oats and barley are linear β-(1→4) linkages separating shorter chain of β-(1→3) structures [4,5,6]. On the other hand, BG of *Candida* spp. (the most common fungus in mammal gut) and bacteria are mainly β-(1→3) backbones with different lengths, with higher and lessor β-(1→6) side chains present in the fungal vs. bacterial BG, respectively [7,8]. Due to the foreignness of these molecules to mammals, all BGs may be categorized as pathogen-associated molecular patterns (PAMPs) if they present to immune cells with appropriate pattern recognition receptors (PRRs) [9,10,11,12]. Although immune responses against PAMPs generally induce inflammatory responses, there are diverse directions of immune activation by different BGs described in the literature. For example, Pachyman and Curdlan, (1→3)-β-D-Glucan from *Poria Cocos* (fungi) and *Alcaligenes faecalis* (Gram-negative bacilli), respectively, can induce harmful proinflammatory immune responses [13,14,15]. Yeast BG (1→3)-β-D-Glucan with β-(1→6)-glucosidic bond-linked side chains and oats or barley (1→3-β-D-Glucan with β-(1→4)-glucosidic linkage elements) [16,17] have been shown to produce beneficial immune modulation and anti-inflammatory effects, respectively [18,19]. Although all BGs are mainly recognized by the Dectin-1 receptor [20,21], the diverse effects of different BGs might partly be due to (i) an ability of BGs to bind to Toll-like receptors (TLR2 and, possibly, TLR4), major PRRs of immune cells [22,23,24], and/or (ii) an impact on gut bacteria, as BGs differentially promote the growth of diverse bacterial groups [25,26,27].

In the human gut, lipopolysaccharide (LPS) and *Candida*-BG are major microbial origin molecules as canonical cell wall components of Gram-negative bacteria and fungi, the most and second-most abundant gut microbes, respectively [28]. With normal gut barrier function, these pathogen molecules are separated from the host’s blood circulation and do not raise systemic immune responses; however, the translocation of these molecules from the gut into the blood circulation (gut translocation) is possible in the circumstance of intestinal barrier injury (leaky gut or gut leakage) [28]. While physiologically transient gut translocation of pathogen molecules may not induce chronic systemic inflammation because of several protective mechanisms [29,30,31], the long duration of gut translocation due to the chronic intestinal barrier defect may occur, with significant, negative sequelae [24,32,33,34]. During episodes of gut barrier injury, BG in serum is correlated with glucans in gut contents, as the oral administration of viable or heat-killed fungi enhances serum BG [27]. This is similar to increased endotoxemia (serum LPS) from Gram-negative bacteria in gut contents [35,36]. Various insults leading to gut permeability are experienced. These may include both normal physiologic (intense exercise and spicy foods) [27,37] and pathogenic conditions (drugs, obesity, autoimmune diseases, sepsis, severe hypoxia, and infection) [10,15,38,39,40,41]. Leaky gut-induced systemic inflammation is possibly involved in several diseases, and the oral administration of different forms of glucans may induce different impacts.

To test this hypothesis, three different BGs, Pachyman (BG with mainly (1→3)-linkages) and very few (1→6)-linkages), whole-glucan particles (WGP) (BG with many (1→6)-glycosidic bonds) [42], and Oat-BG (BG with (1→3) and (1→4)-linkages) [43], were administered in a dextran sulfate-induced leaky gut murine model. Additionally, these BGs with or without LPS were also injected into mice and tested in macrophage culture.

## 2. Results

### 2.1. Pachyman Prominently Increased Serum Cytokines in a DSS-induced Mucositis Mouse Model

**Figure 1:**
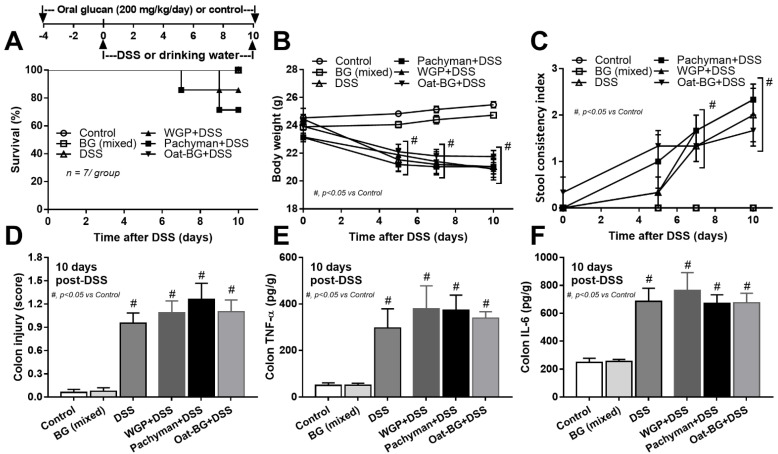
Schema of the experiments (A, upper part) and characteristics of mice with drinking water (Control) with or without oral β-glucan gavage using whole-glucan particles (WGP), Pachyman or Oat glucans (Oat-BG), which are combined into the BG (mixed) group, or dextran sulfate solution with or without several glucans. Survival analysis (**A**), bodyweight alteration (**B**), stool consistency index (**C**), colon injury score from Hematoxylin and Eosin (H&E) staining (**D**), and colon cytokines (TNF-α and IL-6) (**E**,**F**) are shown (*n* = 6–8/group). #, *p* < 0.05 vs. Control as determined by ANOVA with Tukey’s analysis. Data from oral glucan-administered mice using different types of glucans were combined as BG (mixed) due to the nonsignificant difference between groups.

**Figure 2:**
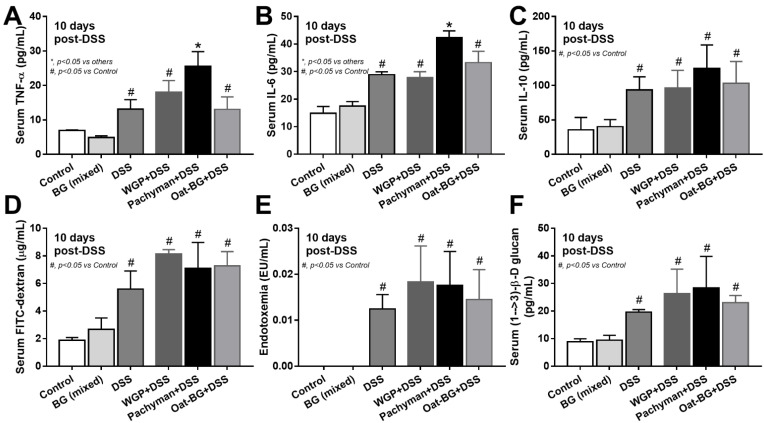
Characteristics of mice with drinking water (Control) with or without oral β-glucan gavage using whole-glucan particles (WGP), Pachyman, or Oat glucans (Oat-BG), which are combined into the BG (mixed) group, or dextran sulfate solution with or without several glucans, as indicated by serum cytokines (TNF-α, IL-6, and IL-10) (**A**–**C**); gut permeability (FITC-dextran assay) (**D**); endotoxemia (**E**); and serum (1→3)-β-D-glucan (BG) (**F**), which are shown (*n* = 6–8/group). #, *p* < 0.05 vs. Control; *, *p* < 0.05 vs. other groups as determined by ANOVA with Tukey’s analysis. Data from oral glucan-administered mice using different types of glucans were combined as BG (mixed) due to the nonsignificant differences between groups.

To test the impact of BG in different forms toward DSS mucositis, BG were orally administered at 4 days prior to DSS administration (Figure 1A, schema). The administration of any forms of BG in DSS mice did not alter the mortality (Figure 1A) or most of the parameters, including weight loss (Figure 1B), stool consistency (Figure 1C), colon damage (histology and colon cytokines) (Figure 1D–F), gut permeability (FITC-dextran assay, endotoxemia, and glucanemia) (Figure 2D–F).

**Figure 3:**
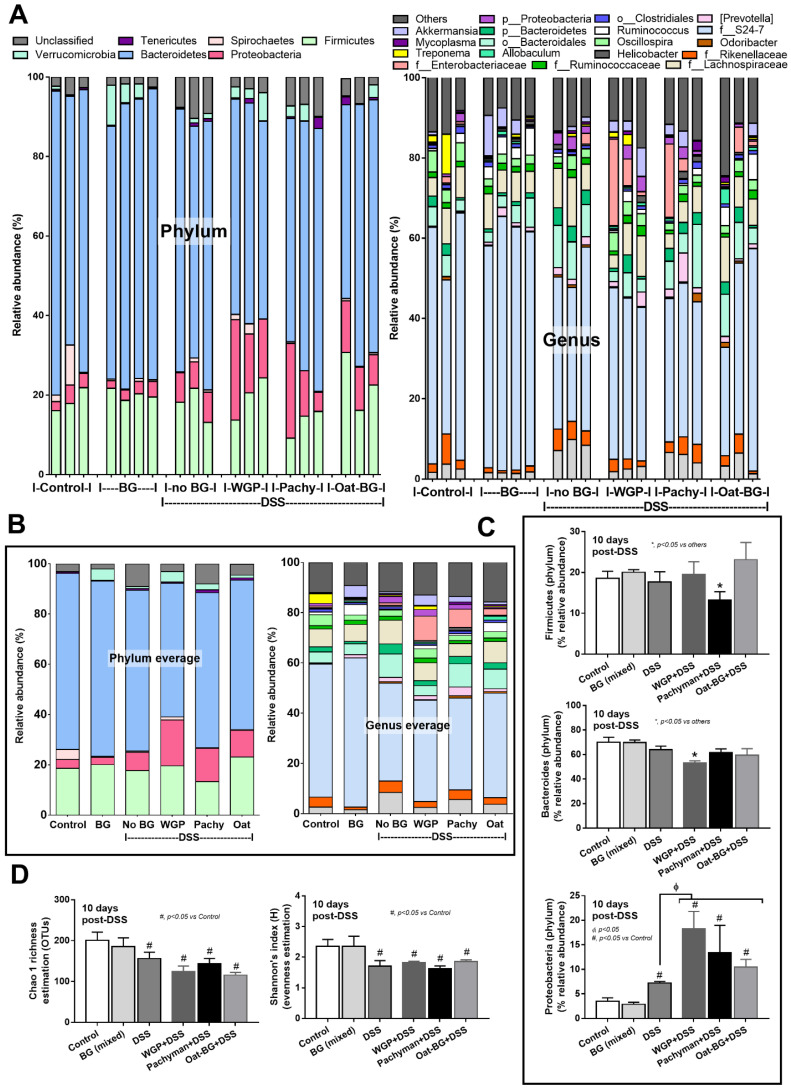
Fecal microbiome analysis from mice. Dextran sulfate solution (DSS) with or without several glucans using whole-glucan particles (WGP), Pachyman, or Oat glucans (Oat-BG) was performed. Meanwhile, in nondrinking water (Control) with oral β-glucans gavage, only one of these glucans was used in each mouse, but the data are combined into the BG (mixed) group. Phylum and genus level determination (**A**), average value of the analysis (**B**), graph presentation in the phylum analysis (**C**), and bacterial diversity (**D**) are shown (*n* = 3–4/group). #, *p* < 0.05 vs. Control; *, *p* < 0.05 vs. other groups; ϕ, *p* < 0.05 vs. the indicated group as determined by ANOVA with Tukey’s analysis. Data from oral glucan-administered mice using different types of glucans were combined as BG (mixed) due to the nonsignificant differences between these groups.

Serum TNF-α and IL-6, but not IL-10 (Figure 2A–C), in Pachyman-administered DSS mice were higher than other groups (Figure 1B–F and Figure 2A–F). No advantage of Oat-BG was demonstrated (Figure 1A–F and Figure 2A–F), possibly due to an inadequate BG dose [40,41] Due to glucan-digestible bacteria and glucan-induced dysbiosis [24], a fecal microbiome analysis was explored. In healthy mice, glucans alone did not alter the microbiome composition compared with the control (Figure 3A–D), while all forms of BG in DSS mice similarly increased the Proteobacteria (pathogenic bacteria) [26], but not microbial diversity, when compared with the non-glucan-supplemented DSS group (Figure 3A–D). In DSS mice, Pachyman profoundly decreased Firmicutes (the most prominent bacteria in gut of healthy hosts) [36], and WGP prominently reduced Bacteroidetes (the most dominant gut Gram-negative bacteria) [36] when compared with non-BG DSS mice (Figure 3C).

**Figure 4:**
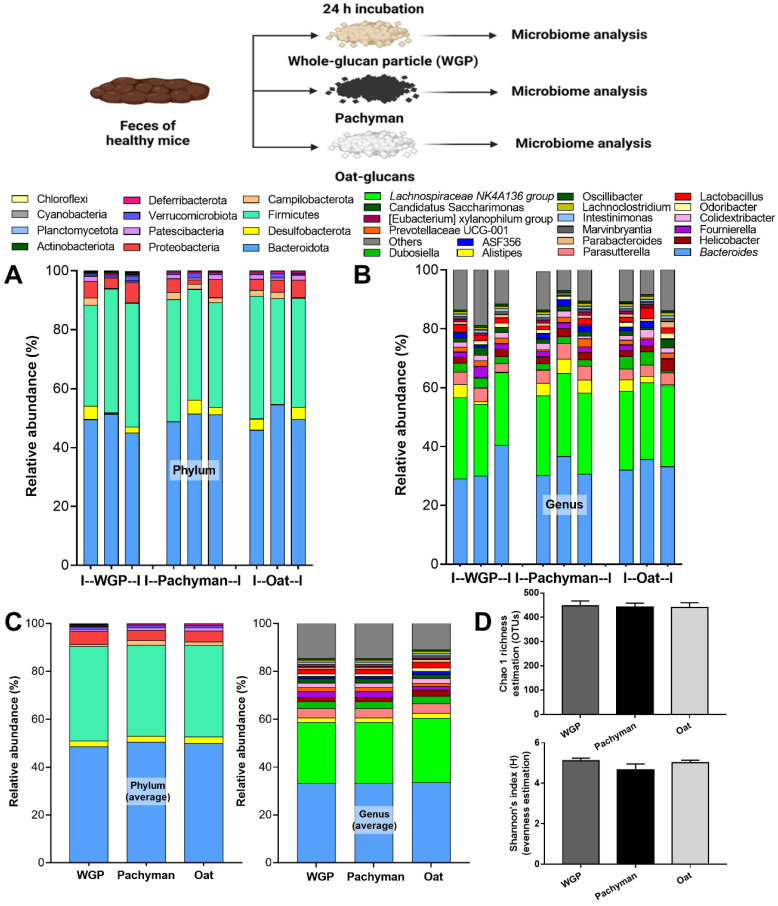
Fecal microbiome analysis of the feces of healthy mice after 24 h of incubation with whole-glucan particles (WGP), Pachyman, or Oat glucans (Oat), as presented in the schema of the experiments, characterized by phylum and genus level determination (**A**,**B**), the average value of the analysis (**C**), and the bacterial diversity (**D**) (*n* = 3/group).

Due to the possible inadequate glucans in the intestines after oral administration in vivo, ex vivo experiments using 24-h BG incubation with feces of healthy mice were performed (Figure 4, schema). However, there was no relative difference of microbiome in the ex vivo fecal experiments with exposure to the different forms of BG (Figure 4A–D).

### 2.2. Enhanced Proinflammatory Effects of Intravenous Administration of LPS Plus Pachyman Than with Other Forms of Glucans

**Figure 5:**
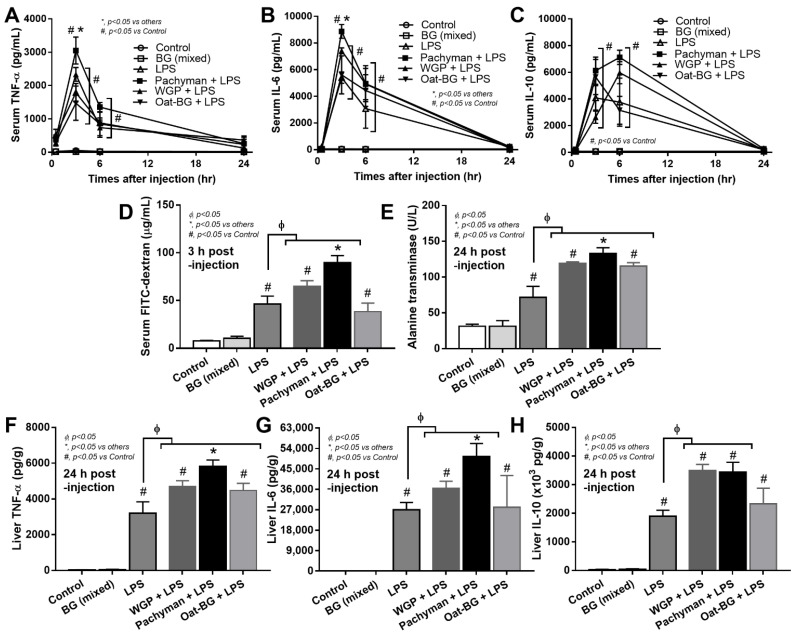
Characteristics of mice with drinking water (Control) with or without oral β-glucans gavage using whole-glucan particles (WGP), Pachyman, or Oat glucans (Oat-BG), which are combined into the BG (mixed) group or dextran sulfate solution with or without several glucans. The alteration in serum cytokines (TNF-α, IL-6, and IL-10); gut permeability (FITC-dextran assay); and liver enzyme (alanine transaminase) (**A**–**E**), together with liver cytokines (TNF-α, IL-6, and IL-10), (**F**–**H**) are shown (*n* = 6–8/group). #, *p* < 0.05 vs. Control; *, *p* < 0.05 vs. other groups; ϕ, *p* < 0.05 vs. the indicated group as determined by ANOVA with Tukey’s analysis. Data from oral glucan-administered mice using different types of glucans were combined as the BG (mixed) due to the nonsignificant different values among the groups with glucan injection alone.

Some parts of BG in the serum of BG-administered DSS mice might be the BG that was orally administered because of the DSS-induced gut barrier defect [9], and these BG might have different effects in mice. To test the impacts of different glucans, BG, with or without LPS, were injected in mice. Glucans alone did not induce serum cytokines. Meanwhile, Pachyman with LPS induced the highest serum inflammatory cytokines (TNF-α and IL-6 but not IL-10) at 3 h post-injection, which were higher than LPS alone or LPS with other glucans (Figure 5A–C). In parallel, Pachyman–LPS induced a more prominent gut permeability (leaky gut), as determined by the FITC-dextran assay, than the other groups. Gut permeability with LPS alone and LPS with non-Pachyman glucans were similar at 3 h post-injection (peak cytokine responses) (Figure 5D). At 24 h post-injection, gut permeability (by FITC-dextran assay) of LPS-administered mice, regardless of the type of glucan, were similar to the control group (data not shown).

Due to hepatic detoxification [44] of blood PAMPs (including glucans), liver injury was explored. Serum alanine transaminase (ALT; liver enzyme) and liver cytokines (TNF-α and IL-6 but not IL-10) were higher in Pachyman–LPS mice than other LPS–BG combinations (Figure 5E–H). At 24 h post-injection, WGP with LPS induced higher ALT and liver cytokines (TNF-α, IL-6, and IL-10), while Oat-BG with LPS induced only higher ALT and liver TNF-α compared to LPS injection alone (Figure 5E–H).

### 2.3. More Profound Synergistic Inflammatory Responses of Macrophages Using LPS Plus Pachyman Compared with LPS Plus Other Glucans

**Figure 6:**
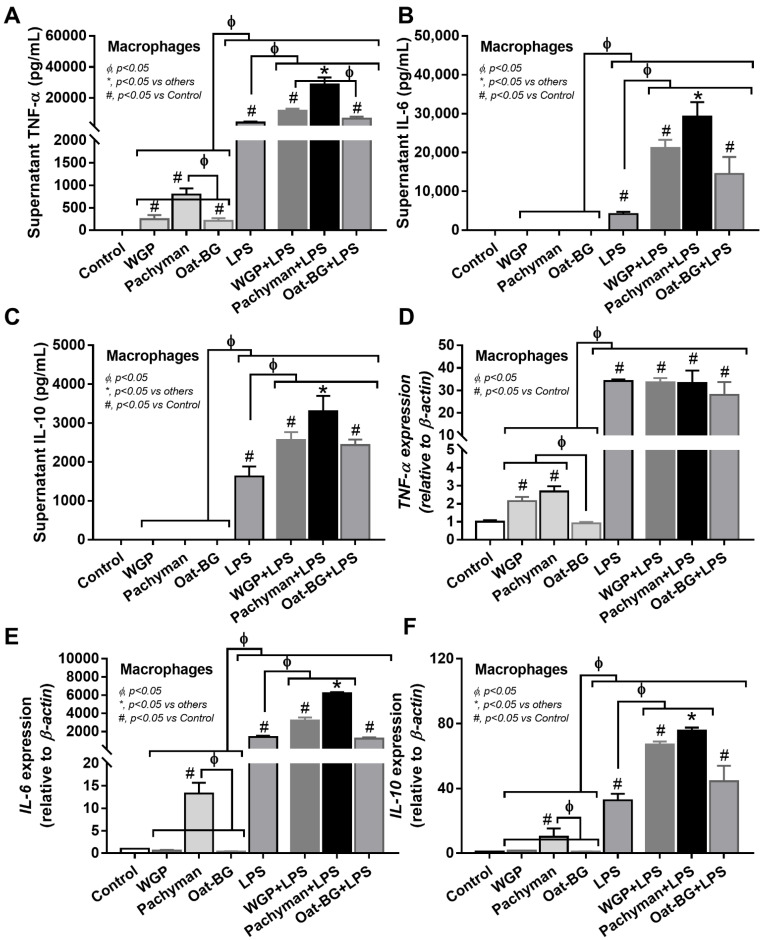
Characteristics of bone marrow-derived macrophages after 24 h of incubation in the control medium (Control) or control medium supplemented with whole-glucan particles (WGP), Pachyman, Oat glucans (Oat-BG), lipopolysaccharide (LPS), and LPS with the different glucans. Supernatant cytokines (TNF-α, IL-6, and IL-10) with gene expression (**A**–**F**) are shown (*n* = 6–8/group). #, *p* < 0.05 vs. Control; *, *p* < 0.05 vs. other groups; ϕ, *p* < 0.05 vs. the indicated group as calculated by ANOVA with Tukey’s analysis. Independent triplicate experiments were performed.

**Figure 7:**
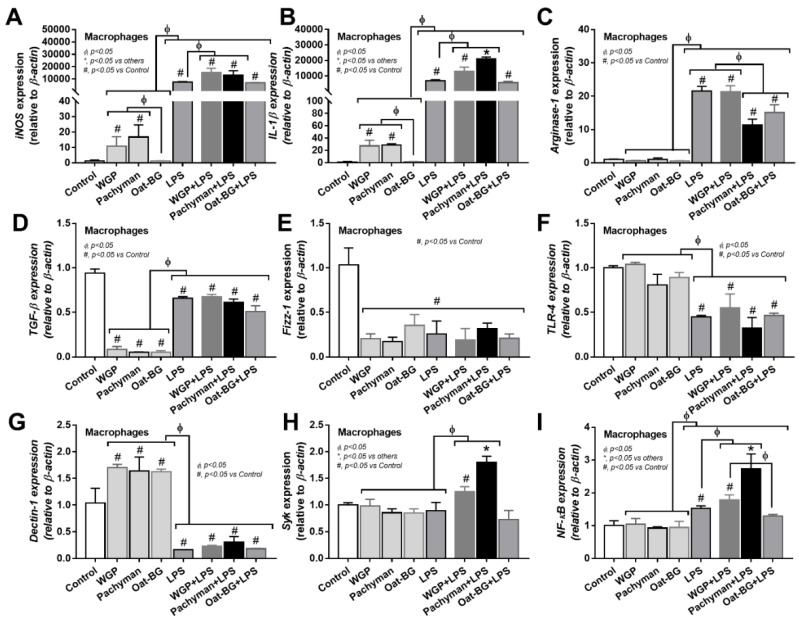
Gene expression characteristics of bone marrow-derived macrophages after 24 h of incubation in the control medium (Control) or supplemented with whole-glucan particles (WGP), Pachyman, Oat glucans (Oat-BG), lipopolysaccharide (LPS), and LPS with the different glucans. Pro- (iNOS and IL-1β) and anti- inflammatory responses (Arginase-1, TGF-β, and Fizz-1) (**A**–**E**) with the signaling genes (TLR-4, Dectin-1, Syk, and NF-κB) (**F**–**I**) are shown (*n* = 6–8/group). #, *p* < 0.05 vs. Control; *, *p* < 0.05 vs. other groups; ϕ, *p* < 0.05 vs. the indicated group as calculated by ANOVA with Tukey’s analysis. Independent triplicate experiments were performed.

In contrast to LPS, all forms of glucans (without LPS) induced quite low levels of supernatant inflammatory cytokines (TNF-α, IL-6, and IL-10) (Figure 6A–C) and the gene expression (TNF-α, IL-6, and IL-10) (Figure 6D–F), which was predominant in Pachyman than other glucans (Figure 6A–F). Likewise, proinflammatory M1 macrophage polarization (iNOS and IL-1β) (Figure 7A–B) and Dectin-1 (Figure 7G) (but not TLR-4, Syk, and NF-κB) displayed a similar pattern among the incubations by different BGs without LPS (Oat-BG induced the lowest iNOS and IL-1β) (Figure 7A–I). Combining Pachyman with LPS strongly induced inflammatory responses, as demonstrated by elevated supernatant cytokines (TNF-α, IL-6, and IL-10) (Figure 6A–C) and the upregulation of several genes (IL-6, IL-10, iNOS, IL-1β, Syk, and NF-κB) (Figure 6E–F and Figure 7A–B,H–I). Differences in anti-inflammatory genes (Arginase-1, TGF-β, and Fizz-1); TLR-4; and Dectin-1 (Figure 7C–G) were not observed between LPS alone vs. LPS with different forms of glucans. Among LPS with glucans, LPS with Oat-BG demonstrated the lowest responses, as most of the parameters (except for supernatant cytokines and Arginase-1) were not different from LPS alone (Figure 6A–F and Figure 7A–I).

**Figure 8:**
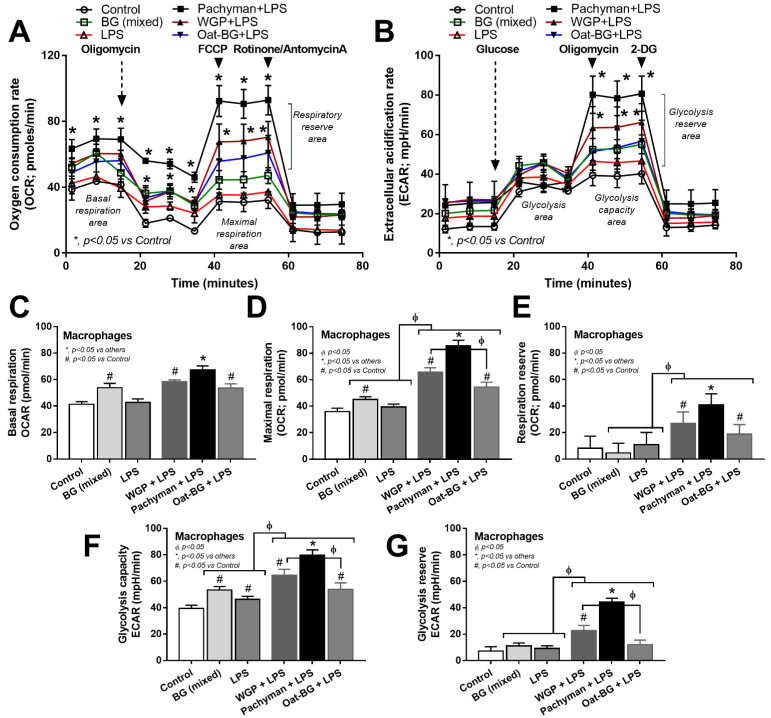
Characteristics of bone marrow-derived macrophages after 24 h of incubation in the control medium (Control) or with or without different forms of β-glucans using whole-glucan particles (WGP), Pachyman, or Oat glucans (Oat-BG), which are combined into the BG (mixed) group, or lipopolysaccharide (LPS) with or without these glucans. Extracellular metabolite flux analysis patterns, including oxygen consumption rate (OCR) of the mitochondrial function (**A**) and extracellular acidification rate (ECAR) of glycolysis activity (**B**) with the graph presentation of mitochondrial (basal respiration, maximal respiration, and respiratory reserve) (**C**–**E**) and glycolysis (glycolysis capacity and glycolysis reserve) (**F**,**G**) parameters, which are shown (*n* = 6–8/group). #, *p* < 0.05 vs. Control; *, *p* < 0.05 vs. other groups; ϕ, *p* < 0.05 vs. the indicated group. ANOVA with Tukey’s analysis was used. Data from oral glucan-administered mice using different types of glucans were combined as BG (mixed) due to the nonsignificant differences between groups and independent triplicate experiments were performed.

Due to the association between cell energy status and cell activities [45], an extracellular energy metabolism metabolite flux analysis was performed. There was an increase in the glycolysis capacity without an alteration of mitochondrial activities (basal respiration, maximal respiration, and respiratory reserve) in macrophages with LPS activation alone, while glucans alone enhanced both glycolysis and mitochondrial activities (Figure 8A–G). Since the glycolysis and respiratory parameters were similar among the activations by any form of BG (data not shown), the groups with glucan activation alone were combined into “BG (mixed)” for easier graphical representation. Interestingly, glucans increased cell energy (Figure 8A–G) despite a subtle proinflammatory activation when compared with LPS stimulation (Figure 6A–F). There was an enhancement in the cell energy status (maximal respiration, respiratory reserve, glycolysis capacity, and glycolysis reserve) in macrophages with LPS plus Pachyman (or WGP but not Oat-BG) compared with LPS alone, with the most prominent in LPS + Pachyman (Figure 8A–G). Only basal respiration and maximal respiration in macrophages with LPS plus Oat-BG were higher than LPS alone (Figure 8A–G), indicating the lower proinflammatory impact of Oat-BG compared with Pachyman and WGP. The profoundly additive cell energy status in LPS + Pachyman-induced macrophages (Figure 8A–G) may be associated with the provision of energy for the robust inflammatory responses [46,47], partly through Syk and NF-κB signaling [11,48].

## 3. Discussion

A variable proinflammatory impact of different β-glucans in DSS-induced gut barrier permeability, possibly through the different potency in the activation of Syk and NF-κB, the common downstream signaling of Dectin-1 and NF-κB, was characterized.

### 3.1. Diversity of Glucans

Glucans (β-glucan; BG) are a group of polysaccharides complex with diverse structures and biological activities that are strongly affected their chemical and physicochemical properties, partly depending on the sources of glucans [49]. While Pachyman and bacterial BG mainly has only a basic linear β-(1→3) structure, WGP (and other BG from mushroom or yeast) consists of a β-(1→3)-D-glucose backbone with the connection point between the backbone at the β-1→6 position (yeast BG is shorter than mushroom BG) [50]. On the other hand, BG from oat and other plant grains (barley and rye) is composed of both β-(1→3) and β-(1→4) glycosidic bonds. Notably, BG that is obtained from bacteria and algae show a linear structure, whereas β-glucan from yeast, mushrooms, oats, and barley exhibits branching structures [51], and the ratio between β-(1→4) and β-(1→3) linkages differ between cereal species [52]. Additionally, different sources of BG consist of different viscosity, and solubility as the solubility of BG from oats and barley is better than wheat-BG [52]. Here, the solubility of the selected BG (Pachyman, WGP, and Oat-BG) is not different. Moreover, BG from the different sources might be differently transformed by gut microbiota, as β-(1→3)/β-(1→4)-glucan can be degraded by *Bacteroides* spp., the most prominent Gram-negative anaerobes in the human gut [53,54]. Despite the extreme diversity of BG, only three forms of BG were selected here as the representative of (i) BG with a predominant linear β-1→3 structure (Pachyman) [55], (ii) BG with a larger (β-1→3)-D-glucose backbone (WGP) with (β-1→6)-linkages [56], and (iii) BG with both (β-1→3)/(β-1→4)-D-glucose backbones (Oat-BG) [57] as a test of the concept studies, due to their frequent use in scientific experiments [58,59].

### 3.2. Oral Administration of Pachyman Caused More Profound Serum Cytokine Elicitation in DSS-Induced Mucositis Mice

Earlier animal model work showed elevated serum β-glucans (BG) after the oral administration of *Candida* (viable or heat-killed) in DSS-induced gut barrier permeability [27], suggesting enhanced gut translocation of intestinal PAMPs. In a situation of less severe gut barrier defect (no diarrhea), oral ingestion of glucans does not enhance the gut translocation of glucans. Similarly, low translocation has been demonstrated in patients with HIV and liver cirrhosis due to a mild degree of gut permeability defect [60]. In this study, DSS (a high molecular weight sulfated polysaccharide) acts as a direct chemical toxin to colonic epithelium resulting in the enhanced gut permeability through a damaged enterocyte tight junction [61] that is severe enough to allow gut translocation of LPS (MW > 8–10 kDa) [62] and BG (MW > 7 kDa) [63]. Normally, passive transport with a healthy gut epithelium is limited to molecules that are smaller than 0.6 kDa [28]. Indeed, Gram-negative bacteria are the main source of intestinal LPS, and BG in the gut is derived from plant contents of food and gut fungi [27,41], and MW of both LPS and BG is too large for passive gut translocation with a healthy gut barrier. Although oral administration of the different forms of BGs did not affect the severity of the gut barrier permeability (FITC-dextran assay, endotoxemia, and serum BG) when compared with DSS mice without oral BGs, orally administered BG might, at least in part, pass through the gut barrier. Although LPS was not directly administered in DSS mice, endotoxemia was spontaneously detectable due to gut permeability damage. In parallel, the administration of glucans, in different forms, into DSS mice possibly initiated LPS plus glucans elevation in serum. Interestingly, Pachyman–DSS mice induced higher serum cytokines (TNF-α and IL-6) than DSS with WGP or Oat-BG, possibly due to the more profound proinflammatory effect of Pachyman than other glucans. A possibility explaining more severe inflammation in Pachyman–DSS mice includes (i) lower gut translocation of Pachyman than other glucans due to higher molecular weight of WGP or Oat-BG over Pachyman [43], (ii) the inflammatory activation might depend on (1→6) glycosidic bonds in WGP and Pachyman, as no (1→6) glycosidic bonds are present in Oat-BG [42,55], and (iii) the variable properties of different glucans in ligating both Dectin-1 and TLR-4 [64]. Notably, the severity of intestinal injury in mice with DSS alone or DSS with BGs was not different, as indicated by weight loss, stool consistency index, histology, and intestinal cytokines, despite a tendency of higher mortality in Pachyman–DSS mice than DSS alone. However, the beneficial effects of Oat-BG [65] and bacterial β-(1→3)-glucan (similar in structure to Pachyman; a high portion of β-(1→3) linked glucose residues) [66,67] or the more severe effect of Zymosan (β-(1→3)/β-(1→6)-glucan from *Saccharomyces cerevisiae*, similar to WGP) [68] upon DSS-mucositis mice were not observed here, possibly due to different doses of administration. Another potential source of differential effects lies with the effects upon the gut microbiome, because different forms of BG might differently affect the gut microbiome composition [69,70]. While no differences in the healthy mice with BG (without DSS), Pachyman + DSS demonstrated the lowest beneficial Firmicute bacteria [40,71], and WGP + DSS produced the lowest Bacteroidetes [40,72,73], and Oat-BG + DSS did not alter the fecal microbiome. These subtle differences of bacterial abundance between BG + DSS vs. DSS alone were accompanied by a similar taxonomic diversity among these groups, which were supported by the ex vivo incubation of mouse feces with different BGs. Hence, Pachyman increased serum cytokines in DSS mice possibly due to the presence of Pachyman BG in blood but not gut dysbiosis nor the severity of intestinal inflammation.

### 3.3. More Profound LPS Synergy with Pachyman, over Other Glucans after Injection in Mice

Although elevated serum BG in DSS mice with oral glucans might be partly due to gut translocation of the administered glucans, there is technical difficulty in the identification of different BGs in blood samples. Then, the inflammatory impact of different forms of BGs was tested by direct injection in mice, and the BGs alone did not induce inflammation [74,75,76]. The injection of BG plus LPS enhanced serum cytokine elicitation (TNF-α and IL-6), with the highest potency observed with LPS combined with Pachyman, followed by LPS + WGP [10], while LPS + Oat-BG only elevated liver IL-10. These data supported a possible different response of LPS combined with different forms of BG [21]. Indeed, Dectin-1, the main BG recognition receptor, is present in several immune cells, including dendritic cells, monocytes, macrophages, neutrophils, B cells, and natural killer cells [77,78]. This multiplicity of responsive cell types suggests in vivo opportunities for the greatly enhanced elicitation of proinflammatory cytokines in settings of co-exposure to PAMPs with different receptors and signal transduction cascades [22,79]. In macrophages, Pachyman and other BG (such as curdlan) demonstrated moderate to high proinflammatory effects among the different forms of BG [59,75], and LPS plus Pachyman induced even more profound inflammation than LPS alone. There was a similar downregulation of TLR-4 and Dectin-1 in LPS alone or LPS with any BG, possibly as a protective response against cell injury from LPS-elicited reactive oxygen species (ROS) [80]. In parallel, the expression of Syk and NF-κB in Pachyman + LPS-activated macrophages were higher than WGP + LPS, while Oat-BG did not have any synergistic effects with LPS. Furthermore, Pachyman + LPS also prominently accelerated cell energy production from both glycolysis and mitochondria that might be necessary during the intensified cytokine production in macrophages when compared with LPS activation alone [47,81]. Hence, the proinflammatory effect of BG on LPS synergy was most prominent with Pachyman, followed by WGP, with the lowest impact found with Oat-BG.

### 3.4. A Possible Impact of Dectin-1 with LPS Co-Stimulation in Macrophage Activation by Pachyman Plus LPS, a Working Hypothesis

**Figure 9:**
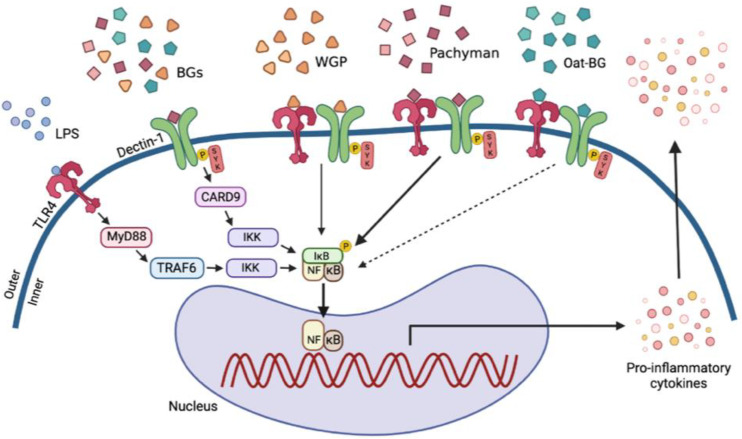
The proposed working hypothesis demonstrates the possible additional proinflammatory impact with lipopolysaccharides (LPS) plus the different forms of (1→3)-β-D-glucan (BGs), including whole-glucan particles (WGP; BGs with 1,6 linkage), Pachyman (BGs without 1→6 nor 1→4 linkages), and Oat β-glucans (Oat-BG; BGs with 1,4 linkage), through the crosslink between Toll-like receptor-4 (TLR-4) and Dectin-1, the pattern recognition receptors for LPS and BGs, respectively, with Spleen tyrosine kinase (Syk), an inhibitor of nuclear factor kappa-B kinase (IKK), and nuclear factor kappa B (NF-κB) as the downstream signals (thickness of the arrows represents the intensity of the activation). While Myeloid differentiation primary response 88 (MyD88) and Syk-Caspase recruitment domain-containing protein 9 (CARD-9) are the main downstream signals of TLR-4 and Dectin-1, respectively [48,64,82], the crosslink of Dectin-1 with TLR-4, by a proper form of glucan (Pachyman), induced stronger signaling through Syk and NF-κB [64]. TRAP-6, Thrombin Receptor Activator for Peptide 6; IκB, I kappa B kinase. The picture is created by BioRender.com (accessed on 1 March 2022).

Despite the Dectin-1-dependent recognition of all forms of BG, the potency of BG downstream signaling by Pachyman seems to be more profound compared to WGP and Oat-BG, as indicated by the level of upregulation of Syk and NF-κB in LPS with Pachyman or WGP compared with LPS activation alone or with Oat-BG. These differences might, partly, be due to differences in the structures of BGs that are associated with the quality of the interaction between the Dectin-1 and TLR-4 signal transduction pathways [48,64,82] (Figure 9). In addition, the possibility of particulate BG ligating both Dectin-1 and TLR4, with synergistic activation enhancement, has been demonstrated in cells transfected with these receptors [79]. Although there is limited exploration of TLR-4 and Dectin-1 ligation by BG, several publications have described TLR4 ligation [83]. We hypothesize that Syk might be responsible for the enhanced LPS-glucans proinflammatory effect, because Syk is (i) directly downstream of Dectin-1 [84], (ii) an alternative (non-MyD88) downstream signal of TLR-4 [85,86], and (iii) one of the major downstream signals after the respective ligation of Dectin-1 and TLR-4 by their respective ligands [64]. It is possible that the β-(1→3) structure of Pachyman, being limited-branching β-(1→3) glucans different from WGP with multiple β-(1→6)-glycosidic branches, Oat-BG, and β-(1→4)-glycosidic linkages, might be responsible for the more effective crosslinks with more profound downstream signals. Additionally, the enhanced cell energy reserve (glycolysis and respiratory reserves) was only demonstrated in WGP and Pachyman but not in Oat-BG, in correlation with the inflammatory activation, possibly to produce the adequate energy for the facilitated inflammatory cell activities [11,87]. More studies on this topic are warranted.

### 3.5. Clinical Perspective

Currently, the impacts of BG on human health are confusing. On one hand, BG from plant grains (oat, barley, cereal, and wheat) demonstrates several health benefits [88,89,90]. On the other hand, gut translocation of BG from intestinal fungi enhances systemic inflammation, indicating a diverse influence of BG in specific conditions [11,34,40]. With the proinflammatory property, Pachyman transforms the anti-inflammatory state around the cancer tissue into proinflammatory conditions that attenuate malignancy [91,92], and WGP incubation induces proinflammatory macrophages [93]. In contrast, Oat-BG exhibits an anti-inflammatory effect in some situations (Crohn’s disease, obesity, and vigorous exercise) [18,19,94,95]. Despite some proinflammatory effects of Oat-BG in our experiments, Oat-BG demonstrated the least inflammatory activity and might be suitable to boost up immune responses [96,97,98,99], induce trained immunity (innate immune memory, the increased innate immune responses after the subsequent activation) [100], or improve gut dysbiosis [101,102,103]. The high viscosity (gelation-liked property) of Oat-BG (and other BG from plant grains) [52] may prevent gut translocation; increase intestinal viscosity; and thereby improve glucose–lipid homeostasis (delayed glucose absorption, decreased bile acid absorption, increased bile acid excretion, and enhanced hepatic cholesterol metabolism) [52]. Due to the high viscosity, Oat-BG might also effectively facilitate the production of short-chain fatty acids from enterocytes that autologously improve enterocyte functions and further inhibit cholesterol synthesis [25]. In contrast, the oral administration of other glucans, especially Pachyman and WGP (BG containing β-(1→3), (1→6)-D-glucan), in conditions with gut barrier defects might enhance systemic inflammation and cause adverse effects in several diseases. On the contrary, Pachyman and WGP might have better benefits on immune activation in some conditions [104], such as sepsis-immune exhaustion (increased susceptibility to secondary infection after sepsis) [105] and cancer-mediated immune responses (the blockage of immune responses by malignant cells) [106]. Overall, oral BG administration in individuals with gut barrier defects (sepsis, severe diarrhea, morbid obesity, and vigorous exercise) [28,29,107] should be done with caution, as only specific glucans might be suitable for specific clinical conditions. It is advised to keep in mind that the presence of any forms of glucans in the blood circulation might be harmful in some hyperinflammatory conditions, because any glucans (including Oat-BG) are foreign to the host and are recognized as pathogen-associated molecules [12]. More studies to match the glucans with their proper clinical use are warranted.

In conclusion, Pachyman and Oat-BG demonstrated the highest and the lowest potency of proinflammatory synergy with LPS, respectively. That is possibly due to the differences in the downstream signaling of the Dectin-1 and TLR-4 signal transduction pathways. Meanwhile, the impact of gut dysbiosis of these glucans was modest in our experiments. The proper selection of glucans to use in specific conditions might be necessary for clinical situations. More studies are warranted.

## 4. Materials and Methods

### 4.1. Animal

The Institutional Animal Care and Use Committee of Chulalongkorn University′s Faculty of Medicine approved the animal study protocol (025/2563), which followed the National Institutes of Health′s (NIH) animal care and use procedure. In the experiments, male 8-week-old mice weighing 20–22 g were purchased from Nomura Siam (Pathumwan, Bangkok, Thailand). The mice were kept in conventional clear plastic cages with free access to water and food containing fat (4.5% *w*/*w*) with the energy content calculated at 3.04 kcal/g (SmartHeart Rodent; Perfect companion pet care, Bangkok, Thailand) in a standard facility with a 12:12 h light/dark cycle at 22 2 °C, 50% relative humidity, and 5 air changes per hours using the heating, ventilation, and air conditioning (HVAC) system.

### 4.2. Dextran Sulfate Solution Model with β-Glucans Administration

Gut permeability (gut leakage) induced by dextran sulfate solution (DSS) with or without administration by the different forms of β-glucans was performed to explore a possible impact of glucans on mucositis. As such, a 40-mg/kg dose of β-glucans [108], using whole-glucan particles (WGP), soluble (1→3)/(1→6)-β-Glucan) extracted from Saccharomyces cerevisiae (InvivoGen, San Diego, CA, USA), Pachyman, soluble (1→3)-β-D-glucan extracted from *Poria cocos* (mushroom) (Megazyme, Bray, Ireland), or Oat-glucans (Oat-BG), (1→3)/(1→4)-β-glucan extracted from *Avena sativa* L. (Oat) (Biosynth Carbosynth, Staad SG, SANKT GALLEN, Switzerland), was orally administered using 18 gauge feeding tubes,1.5 inches in length with a rounded tip mouse oral gavage needle (Sigma-Aldrich, St. Louis, MO, USA) once-daily at 8:00 a.m. from 4 days before DSS (day-4) to 10 days post-DSS. All BG (WGP, Pachyman, and Oat-BG) were gently resuspended in 0.25-M NaOH and kept at 4 °C overnight, with 0.25-M HCl added before being used (both in vivo and in vitro). After that, DSS (Sigma-Aldrich) was diluted at 3% volume by volume (*v*/*v*) into drinking water for 10 days [23]. The stool consistency was semi-quantitatively evaluated using the following scores: 0, normal; 1, soft; 2, loose; and 3, diarrhea, as previously published [23]. At 6 h after the last dose of glucans on the 10th day of DSS, mice were sacrificed by cardiac puncture under isoflurane anesthesia with sample collection. Serum was kept at −80 °C until use, and the organ (ascending colon; 2 cm distally to the cecum) was put in 10% neutral formalin and snap-frozen in −80 °C for histological analysis and organ cytokines, respectively. Notably, the mice were housed individually during the experiments to avoid an impact of allocoprophagy (a habit of ingestion of feces of other mice) on the fecal microbiome analysis [33,38]. Feces were collected post-sacrifice from the cecum.

### 4.3. Lipopolysaccharide with and without Injection of Different β-Glucans Model

To test the impact of different forms of β-Glucans on mice, the glucans were administered with and without LPS in mice. Mice were intraperitoneally (ip) administered with 10 mg/kg of LPS from E. coli 026: B6 (Sigma-Aldrich) with or without intravenous (iv) administration (tail vein injection) of the different glucans at 20 mg/kg. Blood samples were collected through the tail vein at several timepoints, and mice were sacrificed at 24 h post-injection by cardiac puncture under isoflurane anesthesia before sample collection.

### 4.4. Gut Permeability Determination

The gut barrier was determined by fluorescein isothiocyanate-dextran (FITC-dextran) and the spontaneous elevation of lipopolysaccharides (LPS) and (1→3)-β-D-glucan (BG) in the serum (gut translocation) without systemic infection [23]. The detection of FITC-dextran, a gut nonabsorbable molecule at a molecular weight of 4.4 kDa, in the serum after an oral administration indicates gut barrier permeability (leaky gut) [41]. Then, FITC-dextran (Sigma-Aldrich) was orally administered at a concentration of 25 mg/mL in 0.25-mL phosphate buffer solution (PBS) at 3 h before sacrifice, and the serum FITC-dextran was measured by fluorospectrometry (microplate reader; Thermo Scientific, Wilmington, DE, USA). In parallel, serum LPS (endotoxin) BG were measured by HEK-Blue LPS detection (InvivoGen, San Diego, CA, USA) and Fungitell (Associates of Cape Cod, Falmouth, MA, USA). The values of LPS < 0.01 EU/mL and BG <7.8 pg/mL were recorded as 0 due to the limitation of the standard curves.

### 4.5. Histological Analysis, Tissue Cytokines, and Mouse Serum Analysis

Colon histology on Hematoxylin and Eosin (H&E) staining at 200× magnification was semi-quantitatively evaluated [23] based on mononuclear cell infiltration (in mucosa and submucosa); epithelial hyperplasia (epithelial cell in longitudinal crypts); reduction of goblet cell; and epithelial cell vacuolization in comparison with control groups using the following scores: 0: leukocyte <5% and no epithelial hyperplasia (<10% of control): (1) leukocyte infiltration 5–10% or hyperplasia 10–25%, (2) leukocyte infiltration 10–25% or hyperplasia 25–50% or reduced goblet cells (>25% of control), (3) leukocyte infiltration 25–50% or hyperplasia >50% or intestinal vacuolization, and (4) leukocyte infiltration >50% or ulceration. Additionally, localized inflammation in tissue was measured as previously described [34]. Briefly, the samples were weighed, sonicated thoroughly, put into 1-mL PBS per g tissue, centrifuged, and the supernatant was collected for cytokine measurement by ELISA (Invitrogen, Waltham, MA, USA), according to the manufacturer’s protocol. Moreover, serum cytokines (TNF-α, IL-6, and IL-10) and alanine transaminase (ALT) were measured by the enzyme-linked immunosorbent assay (ELISA) (Invitrogen) and EnzyChrom alanine transaminase assay (Bioassay, Hayward, CA, USA), respectively.

### 4.6. Fecal Microbiome Analysis

The method of fecal microbiota analysis followed that of previous publications [9,27,32,109,110]. Briefly, feces (0.25 g per mouse) were used to extract total DNA by a power DNA isolation kit (MoBio, Carlsbad, CA, USA), and the metagenomic DNA quality was evaluated by agarose gel electrophoresis with nanodrop spectrophotometry. Universal prokaryotic forward primer 515F (59-GTGCCAGCMGCCGCGGTAA-39) and reverse primer 806R (59-GGACTACHVGGGTWTCTAAT-39), with an appended 50 Illumina adapter and 30 Golay barcode sequences, were used for 16S rRNA gene V4 library construction. Triplicate polymerase chain reactions (PCRs) were performed, and each reaction mixture consisted of 1 EmeraldAmp1 GT PCR master mix (TaKaRa), 0.2 mM of each primer, and metagenomic DNA (75 ng). A GenepHlow gel extraction kit (Geneaid Biotech Ltd., New Taipei City, Taiwan) was used for purifying 16S rRNA from an agarose gel, and quantification was performed using PicoGreen (Invitrogen, Eugene, OR, USA). Each sample (240 ng) was applied to the MiSeq300 sequencing platform (Illumina, San Diego, CA, USA) with previously described index sequences with Mothur’s standard quality screening operating procedures. To separately determine the impacts of BGs, the ex vivo experiments using feces from heathy mice (combined feces from cecum of 3 mice) at 0.01 g were co-incubated with the different forms of BGs (1 mg) under anaerobic conditions with gas generation sachets (AnaeroPack-Anaero; Mitsubishi Gas Chemical Co. Inc., Tokyo, Japan) at 37 °C for 24 h before performing a microbiome analysis.

### 4.7. Macrophage Experiments

Bone marrow-derived macrophages were prepared from the healthy mice as previously described [15,24,39,111,112]. Briefly, bone marrow from femurs and tibias were collected by 6000 rpm centrifugation at 4 °C and incubated for 7 days with modified Dulbecco’s modified Eagle’s medium (DMEM) with conditioned media of the L929 cell line, containing a macrophage-colony stimulating factor, in a humidified 5% CO_2_ incubator at 37 °C. Then, LPS (*E. coli* 026: B6, Sigma-Aldrich) at 10 ng/mL with or without β-glucans in different forms at 10 µg/mL [79] or media control alone (DMEM) were incubated with macrophages at 1 × 10^5^ cells/well at 37 °C for 24 h before the determination of supernatant cytokines (TNF-α, IL-6, and IL-10) using the ELISA assay (Invitrogen). In parallel, total RNA was prepared by Trizol, quantified by a Nanodrop ND-1000 (Thermo Fisher Scientific), converted into cDNA by the Reverse Transcription System, and performed real-time quantitative reverse transcription-polymerase chain reaction (qRT-PCR) using the SYBR Green system (Applied biosystem, Foster City, CA, USA) for the expression of several genes. The cDNA template and target primers based on the ΔΔCT method (2-∆∆Ct) relative to the β-actin housekeeping gene were conducted. Primers for cytokines (TNF-α, IL-6, and IL-10); M1 proinflammatory macrophage polarization (iNOS and IL-1β); M2 anti-inflammatory macrophage polarization (Fizz-1, Arginase-1, and TGF-β); and inflammatory signals (TLR-4, Dectin-1, and NF-κB) were used (Table 1).

### 4.8. Extracellular Flux Analysis

Extracellular flux analysis with Seahorse XFp Analyzers (Agilent, Santa Clara, CA, USA) was used to determine the energy status of the cells, with the oxygen consumption rate (OCR) and extracellular acidification rate (ECAR) representing mitochondrial function (respiration) and glycolysis activity, respectively [56]. For OCR evaluation, the 24-h stimulated macrophages at 1 × 10^5^ cells/well were incubated for 1 h in Seahorse media (DMEM complemented with glucose, pyruvate, and L-glutamine) (Agilent, 103575–100) before activation by different metabolic interference compounds such as oligomycin, carbonyl cyanide-4-(trifluoromethoxy)-phenylhydrazone (FCCP), and rotenone/antimycin A. On the other hand, glycolysis stress tests were performed using glucose, oligomycin, and 2-deoxy-d-glucose (2-DG) for ECAR measurement. The data were analyzed by Seahorse Wave 2.6 software based on the following equations: (i) respiratory capacity (maximal respiration) = OCR between FCCP and rotenone/antimycin A−OCR after rotenone/antimycin A, (ii) respiratory reserve = OCR between FCCP and rotenone/antimycin A−OCR before oligomycin, (iii) maximal glycolysis (glycolysis capacity) = ECAR between oligomycin and 2-DG−ECAR after 2-DG, and (iv) glycolysis reserve = ECAR between oligomycin and 2-DG–ECAR between glucose and oligomycin.

### 4.9. Statistical Analysis

All data were analyzed by Statistical Package for Social Sciences software version SPSS Statistics 22.0 (SPSS Inc., Chicago, IL, USA) and GraphPad Prism version 7.0 (GraphPad Software Inc., Avenida De La Playa La Jolla, CA, USA). Results were presented as the mean ± standard error (SE). The differences between multiple groups were examined for statistical significance by one-way analysis of variance (ANOVA) with Tukey’s analysis. The survival analysis and timepoint data were determined by the log-rank test and repeated measures ANOVA, respectively. A *p*-value < 0.05 was considered statistically significant.

## Figures and Tables

**Table 1:** List of primers used in the study.

Primers	Forward	Reverse
Tumor necrosis factor-alpha (TNF-α)	5′-CCTCACACTCAGATCATCTTCTC-3′	5′-AGATCCATGCCGTTGGCCAG-3′
Interleukin-6 (IL-6)	5′-TACCACTTCACAAGTCGGAGGc-3′	5′-CTGCAAGTGCATCATCGTTGTTC-3′
Interleukin-10 (IL-10)	5′-GCTCTTACTGACTGGCATGAG-3′	5′-CGCAGCTCTAGGAGCATGTG-3′
Inducible nitric oxide synthase (iNOS)	5′-ACCCACATCTGGCAGAATGAG-3′	5′-AGCCATGACCTTTCGCATTAG-3′
Interleukin-1β (IL-1β)	5′-GAAATGCCACCTTTTGACAGTG-3′	5′-TGGATGCTCTCATCAGGACAG-3′
Arginase-1 (Arg-1)	5′-CTTGGCTTGCTTCGGAACTC-3′	5′-GGAGAAGGCGTTTGCTTAGTTC-3′
Transforming Growth Factor-β (TGF-β)	5′-CAGAGCTGCGCTTGCAGAG-3′	5′-GTCAGCAGCCGGTTACCAAG-3′
Resistin-like molecule-α (Fizz-1)	5′-GCCAGGTCCTGGAACCTTTC-3′	5′-GGAGCAGGGAGATGCAGATGA-3′
Nuclear factor-κB (NF-κB)	5′-CTTCCTCAGCCATGGTACCTCT-3′	5′-CAAGTCTTCATCAGCATCAAACTG-3′
Toll like receptor-4 (TLR-4)	5′-GGCAGCAGGTGGAATTGTAT-3′	5′-AGGCCCCAGAGTTTTGTTCT-3′
Spleen tyrosine kinase (Syk)	5′-CTACTACAAGGCCCAGACCC-3′	5′-TGATGCATTCGGGGGCGTAC-3′
Dectin-1	5′-TCCCGCAATCAGAGTGAAG-3′	5′-GTGCAGTAAGCTTTCCTGGG-3′

## Data Availability

The data is contained within the article.
